# Deciphering the mechanism of Indirubin and its derivatives in the inhibition of Imatinib resistance using a “drug target prediction-gene microarray analysis-protein network construction” strategy

**DOI:** 10.1186/s12906-019-2471-2

**Published:** 2019-03-25

**Authors:** Huayao Li, Lijuan Liu, Jing Zhuang, Cun Liu, Chao Zhou, Jing Yang, Chundi Gao, Gongxi Liu, Changgang Sun

**Affiliations:** 1 First Clinical Medical College, Shandong University of Traditional Chinese Medicine, Jinan, 250014 Shandong People’s Republic of China; 2 Department of Oncology, Affilited Hospital of Weifang Medical University, Weifang, 261031 Shandong People’s Republic of China; 3 Departmen of Oncology, Weifang Traditional Chinese Hospital, Weifang, 261041 Shandong People’s Republic of China

**Keywords:** Indirubin, Derivatives, Imatinib resistance, Drug target prediction, Gene microarray analysis, Protein network construction

## Abstract

**Background:**

The introduction of imatinib revolutionized the treatment of chronic myeloid leukaemia (CML), substantially extending patient survival. However, imatinib resistance is currently a clinical problem for CML. It is very importantto find a strategy to inhibit imatinib resistance.

**Methods:**

(1) We Identified indirubin and its derivatives and predicted its putative targets; (2) We downloaded data of the gene chip GSE2810 from the Gene Expression Omnibus (GEO) database and performed GEO2R analysis to obtain differentially expressed genes (DEGs); and (3) we constructed a P-P network of putative targets and DEGs to explore the mechanisms of action and to verify the results of molecular docking.

**Result:**

We Identified a total of 42 small-molecule compounds, of which 15 affected 11 putative targets, indicating the potential to inhibit imatinib resistance; the results of molecular docking verified these results. Six biomarkers of imatinib resistance were characterised by analysing DEGs.

**Conclusion:**

The 15 small molecule compounds inhibited imatinib resistance through the cytokine-cytokine receptor signalling pathway, the JAK-stat pathway, and the NF-KB signalling pathway. Indirubin and its derivatives may be new drugsthat can combat imatinib resistance.

**Electronic supplementary material:**

The online version of this article (10.1186/s12906-019-2471-2) contains supplementary material, which is available to authorized users.

## Background

Chronic myeloid leukaemia (CML) is a clonal haematopoietic stem cell proliferation-induced myeloproliferative disease [1]. Because of its high heterogeneity and distinct molecular genetic features, it has attracted extensive attention from researchers. The unique cytogenetic features of CML include the Philadelphia chromosome t (9; 22) (q34; q11), forming a BCR-ABL fusion gene; this gene complex encodes a constitutively active form of the BCR–ABL fusion tyrosine kinase protein. The active site of the tyrosine kinase has a binding site for ATP [2]. Most signalling pathways activated by BCR-ABL are involved in promoting the development of cancer in bone marrow cells, including the Ras-MAPK pathway, the Src-Pax-Fak-Rac pathway, the phosphoinositide-3 kinase (PI3K)–Akt pathway, and the JAK-STAT pathway [3–6].

The development of the tyrosine kinase inhibitor (TKI) imatinib represents a milestone in CML treatment. Imatinib binds specifically to the ATP-binding site of BCR-ABL to form a fusion protein complex, locking in the active site [7]. This blocks CML cells whose active sites limit repeated cell growth and cell proliferation, killing the cancer cells. However, TKI treatment is long-term and induces resistance to TKI, often leading to poor clinical outcomes in CML patients. Drug resistance to TKIs is currently a clinical problem for CML. It isvery importantto find a strategy to inhibit imatinib resistance.

Classical traditional Chinese medicine (TCM) in China has been used for thousands of years. Especially in recent years, Chinese medicine has made some progress in the treatment of cancer. For example, Bu-Zhong-Yi-Qi-Decoction (BZYQD) has been reported to induce gastric cancer cell death by nonapoptotic mechanisms and to induce human ovarian cancer cell death by apoptotic mechanisms [8, 9]. Yu Ning, et al., through the combination BZYQD with cisplatin in cisplatin-resistant A549/DDP cells, showed that BZYQD exhibited direct cytotoxic and chemosensitising effects, suggesting that cotreatment with BZYQD and cisplatin might reverse cisplatin resistance by inducing ROS accumulation, activating apoptosis and autophagy by oxidative stress [10]. It was reported that Qingdai acted on a variety of pathways for the treatment of chronic myeloid leukaemia, including cytokine-cytokine receptor interaction, cell cycle, p53 signalling pathway, MAPK signalling pathway, and immune system-related pathways [11]. Indirubin is the most important and valuable compound in Qingdai; it has been determined to be the quality marker of Qingdai in the Chinese Pharmacopoeia (the State Pharmacopoeia Commission of China, 2015). Studies showed that indirubin and its derivatives inhibited imatinib resistance. For example, the AGM130 compound, derived from indirubin, known as a cyclin-dependent kinase inhibitor, was a strong candidate for treating imatinib-resistant CML [12]. Therefore, in this study, we will use the strategy of ‘Drug Target Prediction-Gene Microarray Analysis-Protein Network Construction’ to explore the mechanism of indirubin and its derivatives in inhibiting imatinib resistance.

## Methods

To decipher the mechanisms by which indirubin and its derivatives reverse imatinib resistance, we adopted the following strategies: (1) we Identified the 2D structure of indirubin and its derivatives through data mining; (2) we downloaded GSE2810 from the GEO database and Identified imatinib-resistant DEGs; (3) we predicted targets of indirubin and its derivatives using related databases; (4) we analysed the possible molecular mechanisms of indirubin and its derivatives reversing imatinib resistance; and (5) we verified the results through computer network molecular docking technology.

### Data preparation

#### Identify indirubin and its derivatives

We identified indirubin and its derivatives from two sources: first by searching the PubChem database and then by manually searching PubMed to augment the data. PubChem (https://pubchem.ncbi.nlm.nih.gov) is a public repository for information on chemical substances and their biological activities. As of September 2015, it contained more than 157 million depositor-provided chemical substance descriptions, 60 million unique chemical structures and 1 million biological assay descriptions, covering approximately 10 thousand unique protein target sequences [13]. We searched the PubChem database with “indirubin” as the key word to identify indirubin and its derivatives, downloaded the compound 2D structures and finally downloaded the “smile” format. In order to increase the comprehensiveness of the data, we manually searched the relevant literature in the PubMed database for titles dealing with indirubin derivatives.

#### Identify the putative target of indirubin and its derivatives

It requires much manpower, material and financial resources to Identify targets of indirubin and its derivatives through experimentation. Therefore, we used a computerized virtual platform to screen for targets and then validated the targets by molecular docking or experimental verification. Swiss Target Prediction (http://www.swisstargetprediction.ch/), a web server to accurately predict the targets of bioactive molecules based on a combination of 2D and 3D similarity measures with known ligands, was used to predict the putative targets of the indirubin and its derivatives. Predictions can be carried out in five different organisms, and mapping predictions by homology within and between different species is enabled for close paralogs and orthologs [14]. The “smiles” formats of indirubin and its derivatives were imported into Swiss Target Prediction to predict their putative targets of action. It is noteworthy that the predicted putative target was limited to *Homo sapiens*. In order to improve the reliability of predictions goal, only high-probability targets were selected. All putative targets Identified were sent to the Therapeutic Target Database (TTD) (http://bidd.nus.edu.sg/group/cjttd/, 2015-09-10), the Comparative Toxicogenomics Database (CTD) (http://ctdbase.org/, 2017-12-05) and the PharmGKB (https://www.pharmgkb.org/) to verify whether these putative targets had some connection to CML.

#### Identify imatinib resistance related genes

Gene expression profiling analysis is a useful method with broad clinical application in the identification of tumour-related genes in various types of cancer, from molecular diagnosis to pathological classification, from therapeutic evaluation to prognosis prediction, and from drug sensitivity to neoplasm recurrence [15]. Gene expression profile GSE2810 was downloaded from the Gene Expression Omnibus (GEO) database, GSE2810 data is based on the GPL2531 (Novusgene type 3 Hematology/Oncology TMU 667 array) platform,including 4 samples (2 imatinib-resistant samples and 2 imatinib-sensitive samples). It was submitted by Ohyashiki JH [16]. Quality control of gene expression data was performed using gene-specific probes. The analysis was carried out by using GEO2R, an online analysis tool for the GEO database, based on R language. We applied the analysis to classify the sample into two groups that had similar expression patterns in imatinib-sensitive and imatinib-resistant. We defined genes as differentially expressed (DEGs) when logFC > 1 or logFC < − 1(FC:Fold Change,the difference in the amount of gene expression in the sample). A *p* value < 0.05 was considered statistically significant. To further study the characteristics of DEGs and their functions, we analysed the DEGs with Gene Ontology and KEGG Pathway. Gene Ontology annotates and classifies genes by Molecular Function (MF), biological process (BP) and cellular component (CC). The pvalue of the GO term of the DEGs was calculated, and the most likely related GO term of the differential gene was located [17].KEGG is an online biochemical energy database that contains a set of genomic and enzymatic methods and is an information resource for the systematic analysis of gene functions and associated high-level genomic functions [18]. ClueGo, a plugin for Cytoscape 3.5.1 software, provides systematic and comprehensive biologically functional annotation of high-throughput gene expression [19]. Therefore, ClueGo online tools were employed for GO and KEGG pathway analysis. *P* < 0.05 was considered significant.

### Network construction

Protein-protein network (P-P network). P-P network was built using the relationship between the putative targets of Indirubin and its derivatives and Imatinib resistance related DEGs.

Cytoscape 3.5.1 (http://www.cytoscape.org/) is an open software application for visualizing, integrating, modeling and analyzing interactive networks. All networks are built by it.

### Analysis the protein-protein network

If the degree of a node is more than 2 fold of the median degree of all nodes in a network, such gene hub is believed to play a critical role in the network, and we treat it as major hub. The topological features of the target-target network are analysed by several important topological properties such as degree (the number of links to node) [20], betweenness (the number of shortest paths between pairs of nodes which run through node) [20], closeness(the sum of the distances of node to all other nodes) [20], and K-coreness (a measure of the centrality of node) [21]. The larger a protein’s degree/node betweenness/closeness centrality, the more important that protein is in the PPI network [22]. Subsequently, the targets were screened for topological importance. Then, the major hubs were screened. DAVIDwebserver (https://david.ncifcrf.gov/) was used to perform KEGG pathway enrichment analysis of the main targets.

### Molecular docking simulation

Using computer molecular docking simulation techniques to verify the credibility of the study. SystemsDOCK (http://systemsdock.unit.oist.jp/) were performed to Molecule docking [23]. SystemsDock, a web server for network pharmacology-based prediction and analysis, which permits docking simulation and molecular pathway map for comprehensive characterization of ligand selectivity and interpretation of ligand action on a complex molecular network, the score reported by docK-IN is a negative logarithm of the experimental dissociation/inhibition constant, usually ranging from 0 to 10 (i.e. from weak to strong binding). We conducted molecular docking between the small molecule compounds and their putative targets that are included in the major hubs selected by the P-P network map to evaluate whether indirubin and its derivatives inhibited imatinib resistance.

## Result

### Data preparation

#### Indirubin and 41 derivatives and putative targets

We Identified indirubin and 41 derivatives from the database and downloaded “smiles” format and 2D structures. The putative targets of indirubin and its derivatives were predicted through structural similarities. Indirubin and 41 derivatives and putative targets are shown in Table 1.

**Table 1:**
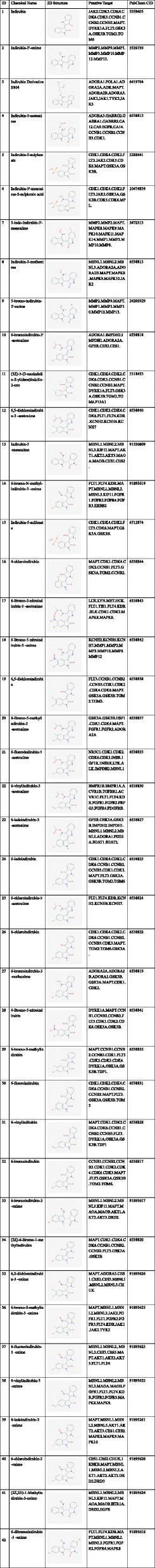
Indirubin and 41 derivatives and putative targets

#### Imatinib resistance related genes

**Fig. 1:**
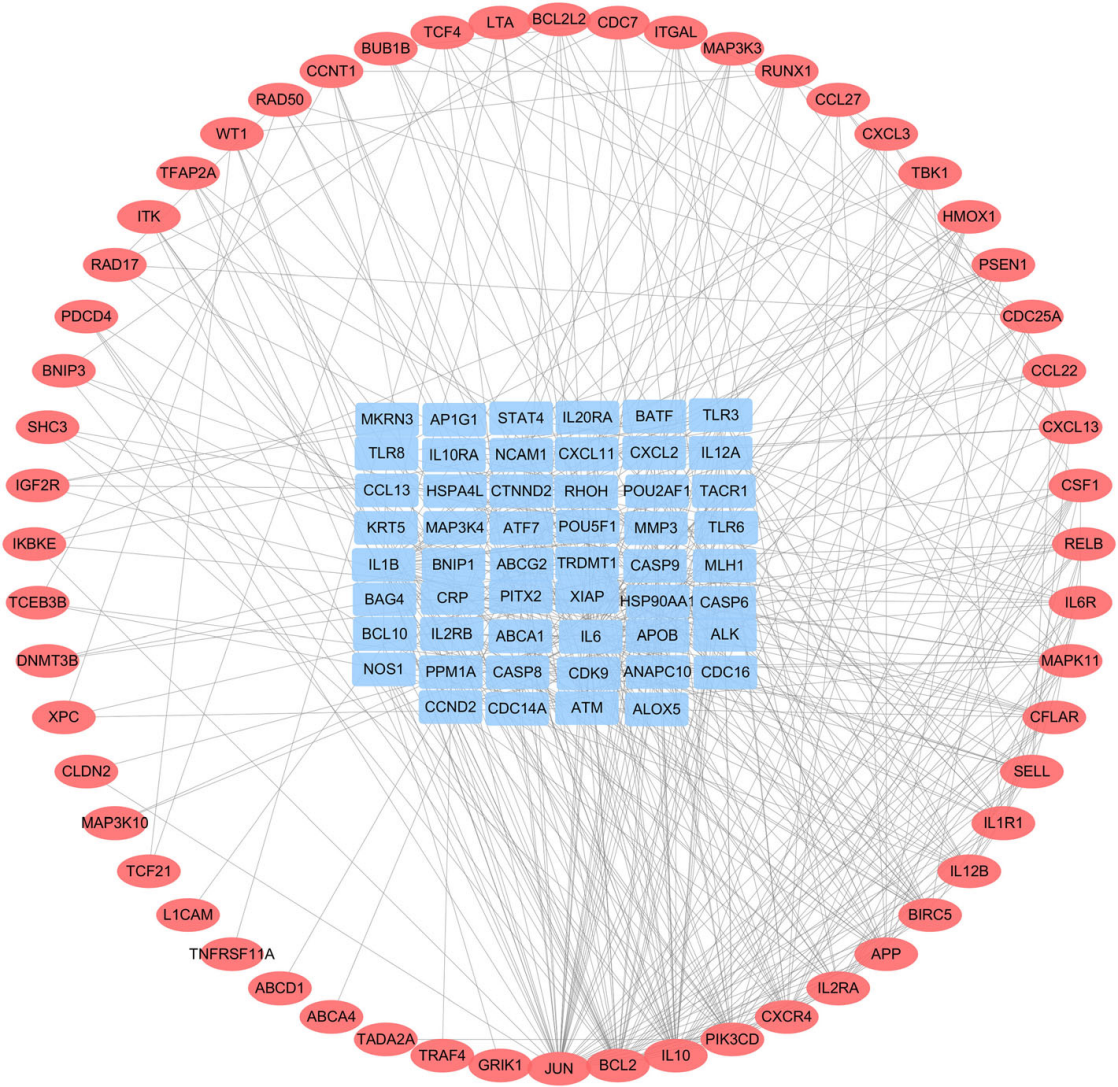
Based on GEO2R analysis, differentially expressed genes of imatinib resistance in chronic myeloid leukemia were Identified from GEO2810(logFC > 1 or logFC < − 1;*P* < 0.05), and a P-P network about DEGswas constructed. The red nodes represent up-regulated differentially expressed genes, and the blue nodes represent down-regulated differentially expressed genes

After gene chip data analysis, we obtained a heat map of the differentially expressed genes of the gene chip G2810 (Additional file 1: Fig. S1), we Identified a total of 125 DEGs with imatinib resistance (Fig. 1), of which 66 were up-regulated and 59 were down-regulated. According to FC,the top 10 significantly up-regulated DEGs and down-regulated DEGs are shown in Table 2. Go analysis and KEGG analysis of DEGs, we found that DEGs of imatinib resistance were closely related to biological processes including immune responses, regulation of protein modification process, regulation of phosphorylation, and regulation of cellular protein metabolic processes. DEGs were mainly involved in cytokine-cytokine receptor interaction pathways.

**Table 2:** The top 10 significantly up-regulated DEGs and down-regulated DEGs

Group	Genesymbol	Gene Description	Fold Change
Upregulated genes	CCL13	C-C motif chemokine 13	9.39035
	MAPK11	Mitogen-activated protein kinase 11	7.52975
	PDCD4	Programmed cell death protein 4	7.43475
	BCL2	Bcl2-associated agonist of cell death	7.22081
	CCL27	C-C motif chemokine 27	6.79919
	TCEB3B	transcription elongation factor B subunit 3B	6.65061
	ANAPC10	anaphase promoting complex subunit 10	6.21695
	IL1R1	interleukin 1 receptor type 1	6.14025
	TCF4	transcription factor 4	5.79877
	TFAP2A	transcription factor AP-2 alpha	5.65156
Downregulated genes	MLH1	MutL homolog 1	−10.7446
	BCL10	B-cell CLL/lymphoma 10	−8.27759
	MAP3K4	mitogen-activated protein kinase kinase kinase 4	−8.1475
	CDK9	cyclin dependent kinase 9	−6.66841
	APOB	apolipoprotein B	−6.5818
	PDGFC	platelet derived growth factor C	−6.62762
	IL10RA	interleukin 10 receptor subunit alpha	−5.64569
	IL12A	interleukin 12A	−5.49548
	CDC14A	cell division cycle 14A	−5.20635
	ALOX5	arachidonate 5-lipoxygenase	−5.20383

CCL13, the first significantly up-regulated chemokine, is a chemotactic factor that attracts monocytes, lymphocytes, basophils and eosinophils [24]. MAPK11, the second significantly up-regulated chemokine, plays an important role in the cascades of cellular responses evoked by extracellular stimuli, including proinflammatory cytokines and physical stress leading to direct activation of transcription factors. The study of Huang J et al. showed that the ERK signalling pathway was more activated in epirubicin treated triple-negative breast cancer (TNBC), possibly contributing to epirubicin resistance, suggesting that the ERK pathway could be used as a novel candidate for targeting therapy in refractory and relapse TNBC [25]. MLH1, the first significantly down-regulated DEG, has been shown to play an important role in haematologic malignancies. The novel mutation was also revealed to be a somatic aberration occurring prior to the initiation of the blast phase in a chronic myelogenous leukaemia (CML) patient. Among the possible MLH1 partners involved in signalling MMR or apoptosis is the proto-oncogene c-MYC, closely associated with cellular proliferation [26]. BCL10, the second significantly down-regulated chemokine, was involved in adaptive immune responses. Proliferation of NIK and IKK cells is promoted by pro-caspase-9 maturation and NF-κB activation.

**Fig. 2:**
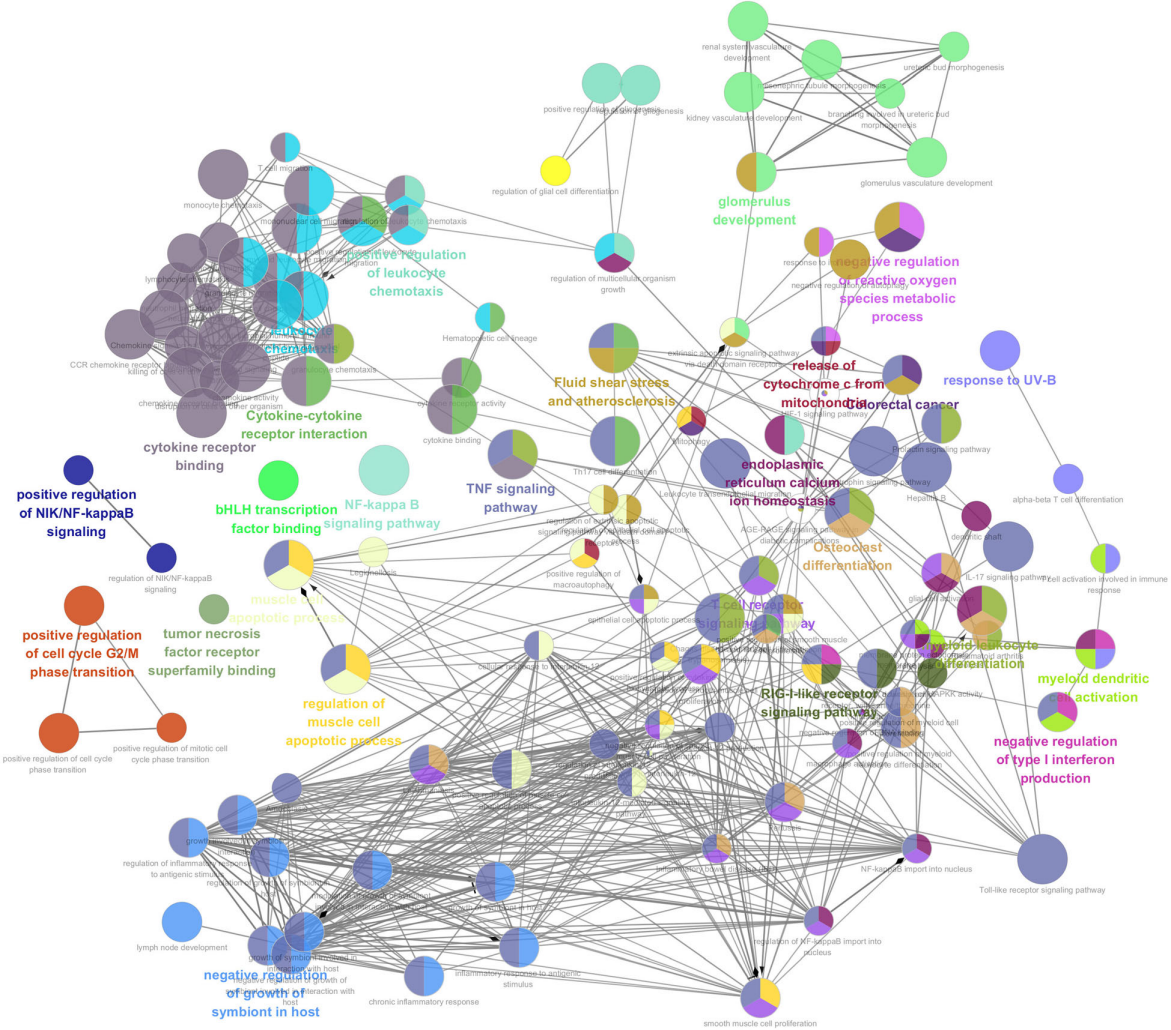
The significantly up-regulated GO categories and enrichment pathways of DEGs(P < 0.05)

**Fig. 3:**
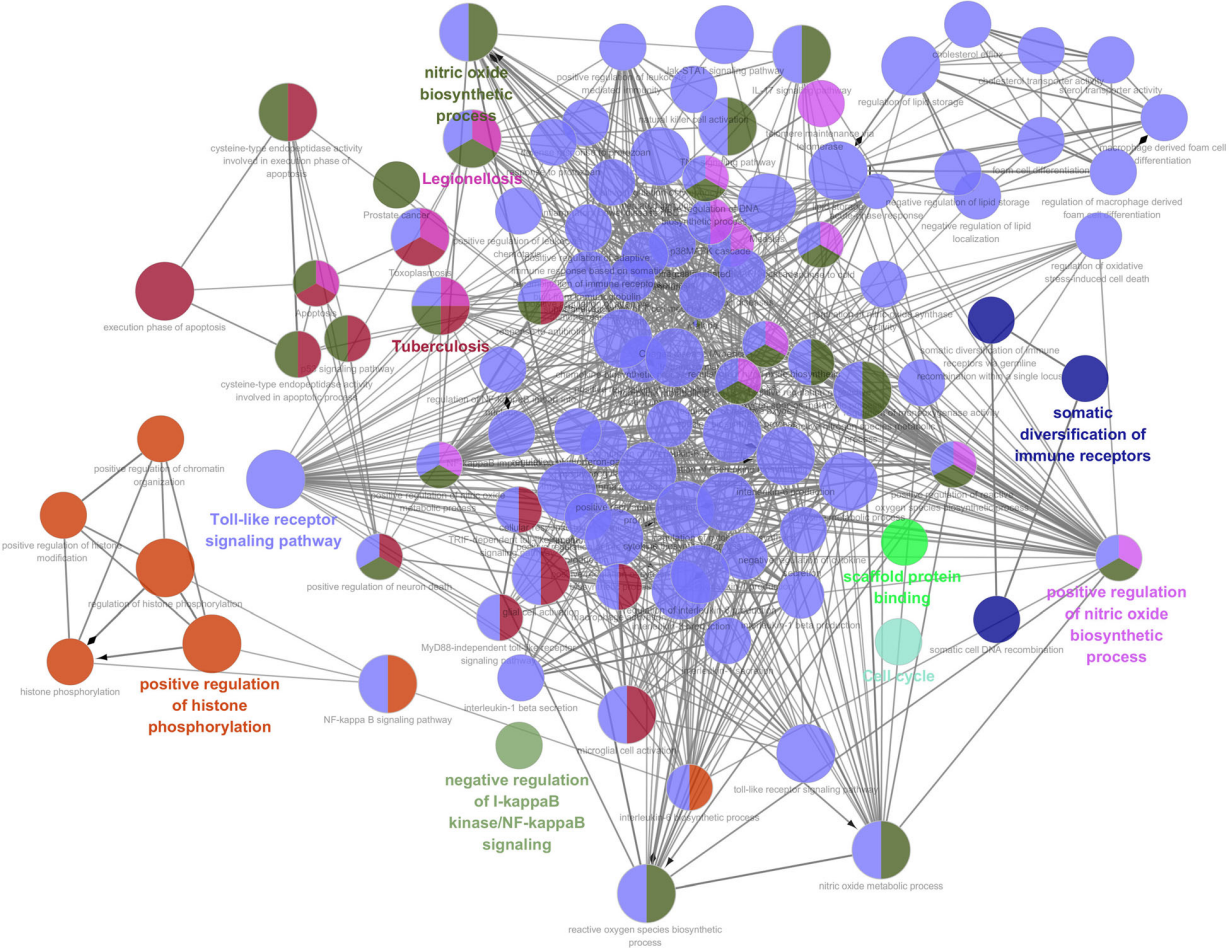
The significantly down-regulated GO categories and enrichment pathways of DEGs(*P* < 0.05)

To further explain the function of differentially expressed genes, we performed functional enrichment analysis of all differential genes based on GO analysis, and performed passway enrichment analysis of all differential genes based on KEGG analysis. we chose significantly up-regulated and down-regulated GO categories based on functional enrichment, The analysis results are shown in Figs. 2 and 3. Through GO analysis, we reached the following conclusions: up-regulated differentially expressed genes were primarily involved in the regulation of cell apoptosis, including immune responses, regulation of apoptosis, regulation of programmed cell death, regulation of cell death, regulation of transcription, cell death, death and DNA binding. The down-regulated DEGs were primarily related to cellular structures, such as cytoplasm, nucleus, extracellular space, positive regulation of transcription from the RNA polymerase II promoter, transcription factor activity and sequence-specific DNA binding growth factor activity. We performed pathway enrichment analysis of differentially expressed genes to Identify the biological pathways. Up-regulated differentially expressed genes were primarily involved in cytokine-cytokine receptor interaction, chemokine signalling pathways, the Toll-like receptor signalling pathway, the neurotrophin signalling pathway, leukocyte transendothelial migration, the MAPK signalling pathway, haematopoietic cell lineage, apoptosis, the T cell receptor signalling pathway and the JAK-STAT signalling pathway. Pathways dramatically altered among down-regulated genes were the cytokine-cytokine receptor interaction, Toll-like receptor signalling pathway, Jak-STAT signalling pathway, pathways in cancer, the NOD-like receptor signalling pathway, apoptosis, cell cycle and the p53 signalling pathway.

**Fig. 4:**
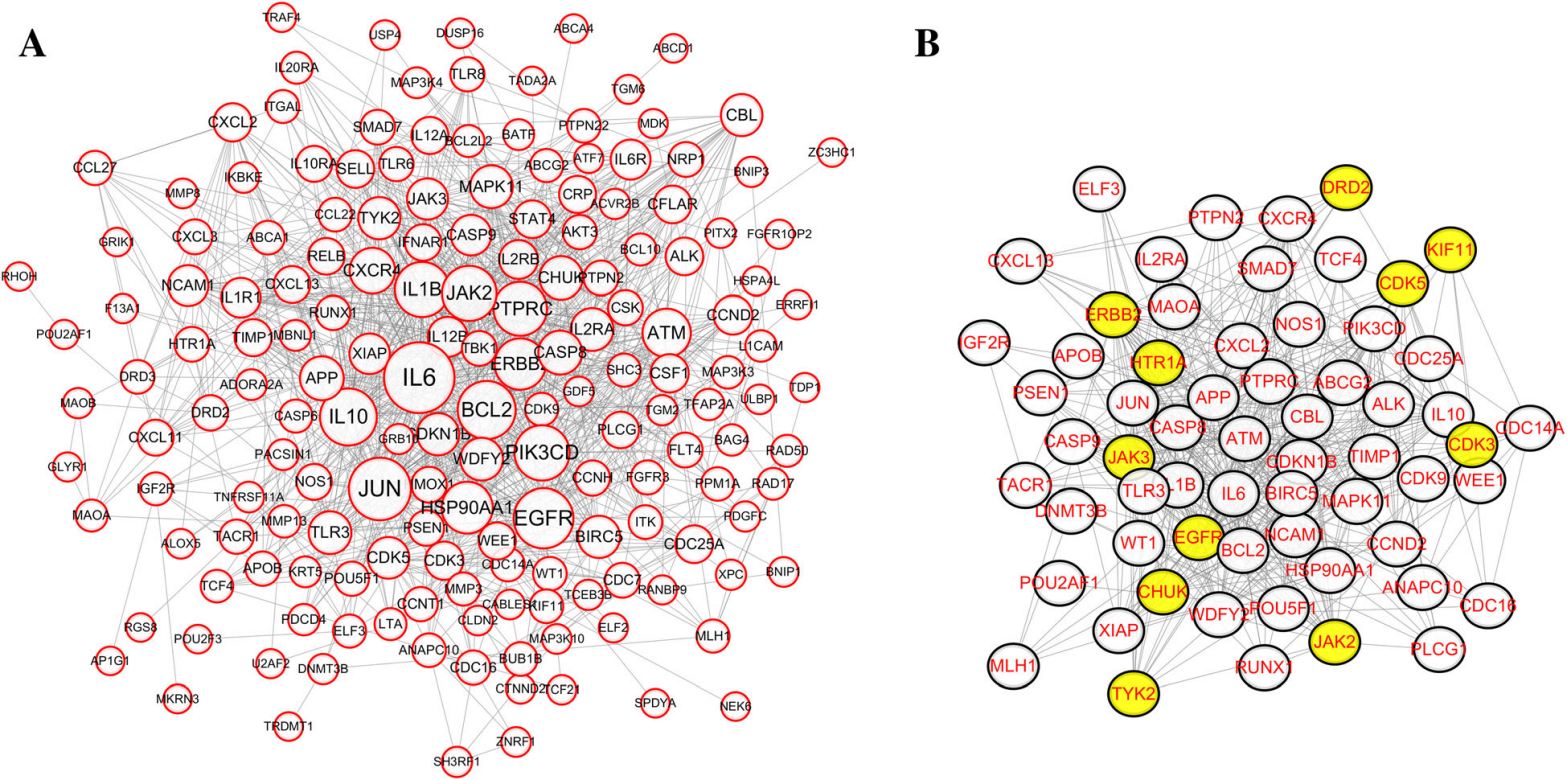
**a** P-P network, a co-expression network of the predicted target of indirubin and its derivatives and imatinib-resistant differentially expressed genes,the size of the node increases as the degree increases; **b** a network of 62 key nodes of the P-P network,the 11 nodes of yellow are not only the predicted targets of indirubin and its derivatives, but also the differentially expressed genes related to imatinib resistance

To identify the relationship between the putative targets of indirubin and its derivatives and DEGs of imatinib resistance, we constructed a P-P network of putative targets and DEGs (Fig. 4). The T-T network consisted of 171 nodes and 1082 edges. The major hubs in the hub interaction network were determined by calculating four features: degree,betweenness,closeness and K-coreness. We showed the major hubs in Fig. 3. After screening, we identified a total of 62 major hubs (Table 3), including 11 (EGFR, JAK2, ERBB2, CHUK, CDK5, KIF11, DRD2, CDK3, HTR1A, JAK3 and TYK2) indirubin and derivative targets and 51 DEGs for imatinib resistance. These 11 major hubs were closely related to DEGs that were resistant to imatinib. Indirubin and its derivatives may inhibit imatinib resistance through the regulation of these genes.

**Table 3:** The 62 major targets information of P-P network

ID	Major target	Uniprot ID	Gene name
MT1	Interleukin-6	P05231	IL6
MT2	Epidermal growth factor receptor	P00533	EGFR
MT3	Transcription factor AP-1	P05412	JUN
MT4	Apoptosis regulator Bcl-2	P10415	BCL2
MT5	Heat shock protein HSP 90-alpha	P07900	HSP90AA1
MT6	Serine-protein kinase ATM	Q13315	ATM
MT7	Tyrosine-protein kinase JAK2	O60674	JAK2
MT8	Phosphatidylinositol 4,5-bisphosphate 3-kinase catalytic subunit delta isoform	O00329	PIK3CD
MT9	Receptor tyrosine-protein kinase erbB-2	P04626	ERBB2
MT10	Baculoviral IAP repeat-containing protein 5	O15392	BIRC5
MT11	Interleukin-1 beta	P01584	IL1B
MT12	Receptor-type tyrosine-protein phosphatase C	P08575	PTPRC
MT13	Mitogen-activated protein kinase 11	Q15759	MAPK11
MT14	Interleukin-10	P22301	IL10
MT15	C-X-C chemokine receptor type 4	P61073	CXCR4
MT16	Amyloid-beta A4 protein	P05067	APP
MT17	Inhibitor of nuclear factor kappa-B kinase subunit alpha	O15111	CHUK
MT18	POU domain, class 5, transcription factor 1	Q01860	POU5F1
MT19	Cyclin-dependent-like kinase 5	Q00535	CDK5
MT20	ATP-binding cassette sub-family G member 2	Q9UNQ0	ABCG2
MT21	Cation-independent mannose-6-phosphate receptor	P11717	IGF2R
MT22	Cyclin-dependent kinase inhibitor 1B	P46527	CDKN1B
MT23	ALK tyrosine kinase receptor	Q9UM73	ALK
MT24	E3 ubiquitin-protein ligase CBL	P22681	CBL
MT25	Substance-P receptor	P25103	TACR1
MT26	Wilms tumor protein	P19544	WT1
MT27	ETS-related transcription factor Elf-3	P78545	ELF3
MT28	G1/S-specific cyclin-D2	P30279	CCND2
MT29	Amine oxidase [flavin-containing] A	P21397	MAOA
MT30	Metalloproteinase inhibitor 1	P20414	TIMP1
MT31	Kinesin-like protein KIF11	P52732	KIF11
MT32	Cell division cycle protein 16 homolog	Q13042	CDC16
MT33	Nitric oxide synthase, brain	P29475	NOS1
MT34	DNA (cytosine-5)-methyltransferase 3B	Q9UBC3	DNMT3B
MT35	1-phosphatidylinositol 4,5-bisphosphate phosphodiesterase gamma-1	P19174	PLCG1
MT36	POU domain class 2-associating factor 1	Q16633	POU2AF1
MT37	E3 ubiquitin-protein ligase XIAP	P98170	XIAP
MT38	Anaphase-promoting complex subunit 10	Q9UM13	ANAPC10
MT39	Runt-related transcription factor 1	Q01196	RUNX1
MT40	WD repeat and FYVE domain-containing protein 2	Q96P53	WDFY2
MT41	M-phase inducer phosphatase 1	P30304	CDC25A
MT42	D(2) dopamine receptor	P14416	DRD2
MT43	CASP8 and FADD-like apoptosis regulator	O15519	CASP8
MT44	Cyclin-dependent kinase 3	Q00526	CDK3
MT45	Tyrosine-protein phosphatase non-receptor type 2	P17706	PTPN2
MT46	DNA mismatch repair protein Mlh1	P40692	MLH1
MT47	Wee1-like protein kinase	P30291	WEE1
MT48	Neural cell adhesion molecule 1	P30291	NCAM1
MT49	Caspase-9	P55211	CASP9
MT50	Toll-like receptor 3	O15455	TLR3
MT51	C-X-C motif chemokine 2	P19875	CXCL2
MT52	5-hydroxytryptamine receptor 1A	P08908	HTR1A
MT53	Mothers against decapentaplegic homolog 7	O15105	SMAD7
MT54	Transcription factor 4	P15884	TCF4
MT55	Tyrosine-protein kinase JAK3	P52333	JAK3
MT56	Interleukin-2 receptor subunit alpha	P01589	IL2RA
MT57	Non-receptor tyrosine-protein kinase TYK2	P29597	TYK2
MT58	Dual specificity protein phosphatase CDC14A	Q9UNH5	CDC14A
MT59	Cyclin-dependent kinase 9	P50750	CDK9
MT60	Presenilin-1	P49768	PSEN1
MT61	Apolipoprotein B-100	P04114	APOB
MT62	C-X-C motif chemokine 13	O43927	CXCL13

We manually screened out small molecule compounds that affected 11 major hubs in the putative target. After screening, a total of 15 small molecule compounds affected these putative targets, including 1, 3, 4, 5, 6, 8, 11, 14, 21, 24, 26, 33,36, 40, 41. These derivatives may all inhibit imatinib resistance. To further verify this conclusion, we evaluated docking of small molecule compounds and their putative targets that were included in the major hubs. The docking results are shown in Table 4.

**Table 4:**
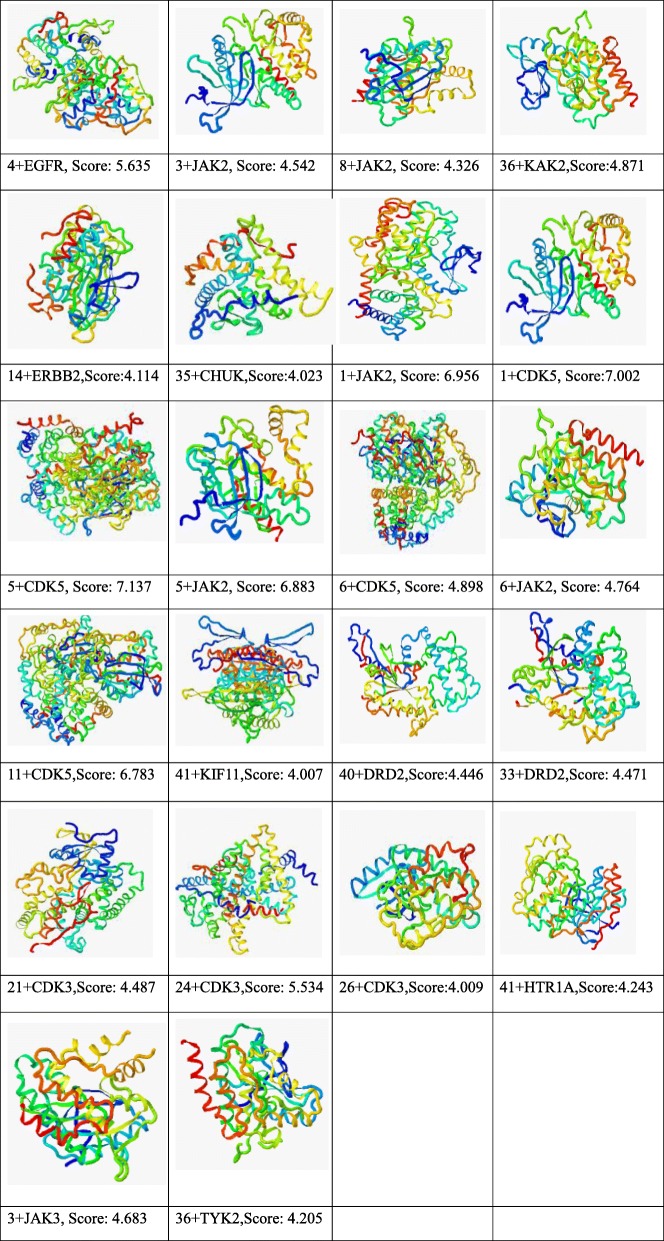
The docking results of molecule compounds and their putative targets. ‘4 + EGFR’ represents the molecular docking of the indirubin derivative numbered 4 with EGFR, and Score represents the score Identified by molecular docking

## Discussion

Qingdai is a traditional Chinese medicine used to treat CML; it is the major active TCM of Qing-Huang-San [27], a Chinese traditional medicine used for the treatment of CML symptoms. It has been widely used in China and has achieved good clinical results. Indirubin is the major active component of Qingdai. Numerous studies have shown that indirubin and its derivatives not only promote apoptosis of CML cells but also inhibit imatinib resistance, including indirubin, indirubin derivative E804, and indirubin-3-acetoxime [28–30]. The exact mechanism of action remains unclear. Therefore, We used the Drug Target Prediction-Gene Microarray Analysis-Protein Network Construction model to investigate the mechanism by which indirubin and its derivatives inhibit imatinib resistance. Various methods, including indirubin derivative screening, drug target search screening, gene chip analysis, network construction, network target analysis, and molecular docking were combined to perform this study. A total of 42 small-molecule compounds were collected and predicted for putative targets. A total of 125 DEGs were selected for imatinib resistance. A total of 15 small-molecule compounds were found to inhibit imatinib resistance by 11 related genes. In our research, data mining of existing databases allows for the objective and rapid discovery of associations and identification of potential drug targets to facilitate the discovery of drugs that inhibit imatinib resistance.

CML is a major haematological malignancy. Imatinib is one of the primary drugs for the treatment of chronic myelogenous leukaemia; however, due to the resistance to imatinib, we were forced to study new drugs to inhibit the resistance to imatinib [31]. Drug resistance involves multiple steps and multiple genes. Therefore, various studies have analysed the differences in gene expression in imatinib-resistant and non-resistant genes by genomic microarrays. In the present study, we performed Go analysis and KEGG analysis on 125 differentially expressed genes and found that the resistance to imatinib was closely related to the following signalling pathways: (1) cell cycle, cell transcription, proliferation, apoptosis, and angiogenesis-related pathways; (2) cytokine-cytokine receptor interaction and chemokine signalling pathways; (3) cancer system related pathways, including pathways in cancer, the p53 signalling pathway and Jak-STAT signalling; (4) the immune system signalling pathway, the T cell receptor signalling pathway, the Toll-like receptor signalling pathway and the NOD-like receptor signalling pathway.

By analysing DEGs, we found that individual genes can serve as biomarkers for imatinib resistance. In up-regulated DEGs, CCL-13, the most significant up-regulated DEGs, is a chemokine that induces eosinophilic chemicals [32]; it can be involved in the interaction between haematopoietic stem cells and the bone marrow microenvironment [33]. In addition, the cytokine-cytokine receptor and chemokine signalling pathways involved in CCL-13 are important pathways involved in imatinib resistance. MAPK11 is the second most prominently expressed gene in the up-regulated differentially expressed genes for imatinib resistance, and MAPK11 is an important constituent gene of the MAPK signalling pathway and is involved in the regulation of various angiogenesis-related diseases [34]. The MAPK signalling pathway is significantlyaugmentedafter imatinib resistance and may be closely related to imatinib resistance. MAPK11 is also involved in up-regulating multiple regulatory pathways for DEGs, including the Toll-like receptor signalling pathway and leukocyte transendothelial migration. PIK3CD is involved in almost all pathways involved in the up-regulation of differentially expressed genes and is significantly augmentedin the course of imatinib resistance. Mesenchymal stem cells (MSC) from BM of chronic myeloid leukaemia (CML) patients on interaction with CML cells or its secreted factors, secreted high levels of IL6, providing a survival advantage to CML cells from imatinib-induced apoptosis [35]; Thus, IL6 may contribute to CML immune escape. Moreover, IL6 is involved in the cytokine-cytokine receptor interaction, the Jak-STAT signalling pathway, and pathways in cancer; therefore, it is closely related to imatinib resistance.

In the down-regulated DEGs, CASP8, an apoptosis-related factor, is an important apoptosis-related gene. Investigators used quantitative PCR to study apoptotic gene expression profile before and after imatinib treatment; they suggested that apoptosis-related gene expression profiles were associated with primary resistance to imatinib [36]. IL12A enhances cellular immunity in the treatment of CML. Studies have shown that immunotherapy enhanced the efficacy of imatinib, and low expression of IL12A led to immune escape of CML cells [37]. Therefore, CCL13, MAPK11, PIK3CD, IL6, CASP8, and IL12A play an important role in the process of imatinib resistance and can be used as biomarkers for imatinib resistance.

To elucidate the relationship between indirubin and its derivatives and imatinib resistance, we constructed a P-P network [38]. By analysing the P-P network, we found that there was a close relationship between the putative target of indirubin and its derivatives and DEGs of imatinib resistance. Through screening, we characterised a total of 11 putative targets [39]. Indirubin and its derivatives may inhibit imatinib resistance through these 11 putative targets. Based on 11 putative targets, we screened 15 small molecule compounds.

Among the 11 putative targets, gefitinib, an EGFR inhibitor, was tested in combination with imatinib in K562 CML cell line using MTT cell proliferation assay and was found to have a synergistic antiproliferative activity; EGFR inhibits or reverses imatinib resistance by enhancing the ability of imatinib to bind at the ATP-binding site of Bcr-Abl kinase [40]. The study found that JAK2 and JAK3 had antiproliferative effects on imatinib-resistant BCR-ABL(+) cells [41], and the administration of imatinib plus a JAK inhibitor reduced expression of stem cells markers, enhancing the antitumour effects of imatinib in CML cells [42]. Human ERBB2 is a proto-oncogene that codes for the erbB-2 epithelial growth factor receptor [43]. CHUK plays an important role in the NF-κB signalling pathway; indirubin and its derivatives inhibited CML cell proliferation by inhibiting CHUK activation of the NF-κB signalling pathway [44]. A study showed that NF-κB represents a potential target for molecular therapies in CML [45]. KIF11 inhibited cell proliferation by blocking the cycle of CML cells. The data showed that KIF11 was overexpressed in BCR-ABL+ CML cells and may become a novel treatment agent for patients with CML [46]. Administration of the imatinib plus JAK inhibitor reduces the expression of stem cell markers, such as ABCG2 and ALDH1A1. Blocking JAK3 with imatinib and JAK3 inhibitors may represent a new therapeutic strategy for eradicating LSCs and preventing CML recurrence [47].

We Identified a total of 15 small-molecule compounds that showed potential inhibition or reversal of resistance to imatinib. Active indirubins might inhibit T315I Abl kinase through unprecedented binding to both active and Src-like inactive conformations [30]. The AGM130 compound is derived from indirubin; data showed that the AGM130 compound efficiently decreased the viability of CML-derived K562 cells. Moreover, this compound also efficiently decreased the viability of imatinib-resistant CML cells in in vitro and in vivo systems [5]. E804, the most potent in indirubin derivative, blocked Stat5 signalling in human K562 CML cells, inhibiting the SFK/Stat5 signalling pathway downstream of Bcr-Abl, leading to apoptosis of K562, KCL-22 M and primary CML cells [48]. In the present study, we Identified small-molecule compounds of indirubin and its derivatives that could potentially inhibit imatinib resistance through drug target prediction, gene microarray analysis, and network construction, accelerating the discovery of new drugs for the treatment of imatinib resistance.

Finally, we used computer simulation techniques to dock selected small-molecule compounds to putative targets, and docking scores showed meaningful results, indicating that our series of strategies can achieve the desired results.

## Conclusion

Definition of a potential drug target is an important first step in the process of drug discovery and drug design. Gene microarray analysis and protein network mapping can be key tools for identification of the factors that play a role in disease progression and thus are the potential drug targets. Subsequently, molecular docking experiments in silico can be used to predict putative interaction of small molecule compounds with the identified targets. In this study, based on the above methods, the mechanism of action of indirubin and its derivatives in inhibiting or reversing the resistance to imatinib was explored, and biomarkers and novel therapeutic targets that inhibited the resistance to imatinib were discovered. We validated experimental results by computerized molecular docking techniques. A limitation of this study was that the results were initially verified by computer simulation, and further verification can be achieved through experimental research.

## Additional file

Additional file 1: **Figure S1.** Heat maps of differentially expressed genes associated with imatinib resistance (we selected 100 genes with the most significant differential expression) (*P* < 0.05). The color from blue to red shows a trend from low to high expression. (JPG 298 kb)

## Abbreviations

BP: Biological process
BZYQD: Bu-Zhong-Yi-Qi-Decoction
CC: Cellular component
CML: Chronic myeloid leukaemia
CTD: Comparative toxicogenomics database
DAVID: The Database for Annotation, Visualization and Integrated Discovery
DEGs: Differentially expressed genes
GEO: Gene expression omnibus
Go: Gene ontology analysis
KEGG: Kyoto encyclopedia of genes and genomes
MF: Molecular function
P-P network: Protein-protein network
TCM: Traditional Chinese medicine
TKI: Tyrosine kinase inhibitor
TTD: Therapeutic target database
