# Supplementary figures and images for: Deciphering the mechanism of Indirubin and its derivatives in the inhibition of Imatinib resistance using a “drug target prediction-gene microarray analysis-protein network construction” strategy
Source: BMC Complement Altern Med. 2019 Mar 25;19:75. doi: 10.1186/s12906-019-2471-2 (PMC6434895; doi:10.1186/s12906-019-2471-2)

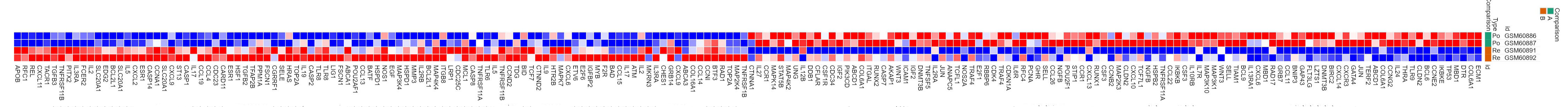

Supplement: Supplementary file 1 — Figure S1. Heat maps of differentially expressed genes associated with imatinib resistance (we selected 100 genes with the most significant differential expression) (P < 0.05). The color from blue to red shows a trend from low to high expression. (JPG 298 kb) [file 12906_2019_2471_MOESM1_ESM.jpg]
